# Data-Augmented Deep Learning for Downhole Depth Sensing and Validation

**DOI:** 10.3390/s26030775

**Published:** 2026-01-23

**Authors:** Si-Yu Xiao, Xin-Di Zhao, Tian-Hao Mao, Yi-Wei Wang, Yu-Qiao Chen, Hong-Yun Zhang, Jian Wang, Jun-Jie Wang, Shuang Liu, Tu-Pei Chen, Yang Liu

**Affiliations:** 1 Micro-nano Integrated Circuit and System Laboratory, University of Electronic Science and Technology of China, Chengdu 611731, China; xiaosiyu@alu.uestc.edu.cn (S.-Y.X.); wangjunjie@uestc.edu.cn (J.-J.W.); liushuang@uestc.edu.cn (S.L.); 2 Southwest Branch of China National Petroleum Corporation Logging Co., Ltd., Chongqing 401100, Chinazhaoxind_cj@cnpc.com.cn (X.-D.Z.); 3 School of Electrical and Electronic Engineering, Nanyang Technological University, Singapore 639798, Singapore; echentp@ntu.edu.sg

**Keywords:** casing collar locator, data augmentation, deep learning, downhole positioning, engineering applications, intelligent sensors, pattern recognition, signal processing

## Abstract

Accurate downhole depth measurement is essential for oil and gas well operations, directly influencing reservoir contact, production efficiency, and operational safety. Collar correlation using a casing collar locator (CCL) is fundamental for precise depth calibration. While neural network has achieved significant progress in collar recognition, preprocessing methods for such applications remain underdeveloped. Moreover, the limited availability of real well data poses substantial challenges for training neural network models that require extensive datasets. This paper presents a system integrated into a downhole toolstring for CCL log acquisition to facilitate dataset construction. Comprehensive preprocessing methods for data augmentation are proposed, and their effectiveness is evaluated using baseline neural network models. Through systematic experimentation across diverse configurations, the contribution of each augmentation method is analyzed. Results demonstrate that standardization, label distribution smoothing (LDS), and random cropping are fundamental prerequisites for model training, while label smoothing regularization (LSR), time scaling, and multiple sampling significantly enhance model generalization capabilities. Incorporating the proposed augmentation methods into the two baseline models results in maximum F1 score improvements of 0.027 and 0.024 for the TAN and MAN models, respectively. Furthermore, applying these techniques yields F1 score gains of up to 0.045 for the TAN model and 0.057 for the MAN model compared to prior studies. Performance evaluation on real CCL waveforms confirms the effectiveness and practical applicability of our approach. This work addresses the existing gaps in data augmentation methodologies for training casing collar recognition models under CCL data-limited conditions, and provides a technical foundation for the future automation of downhole operations.

## 1. Introduction

**Figure 1:**
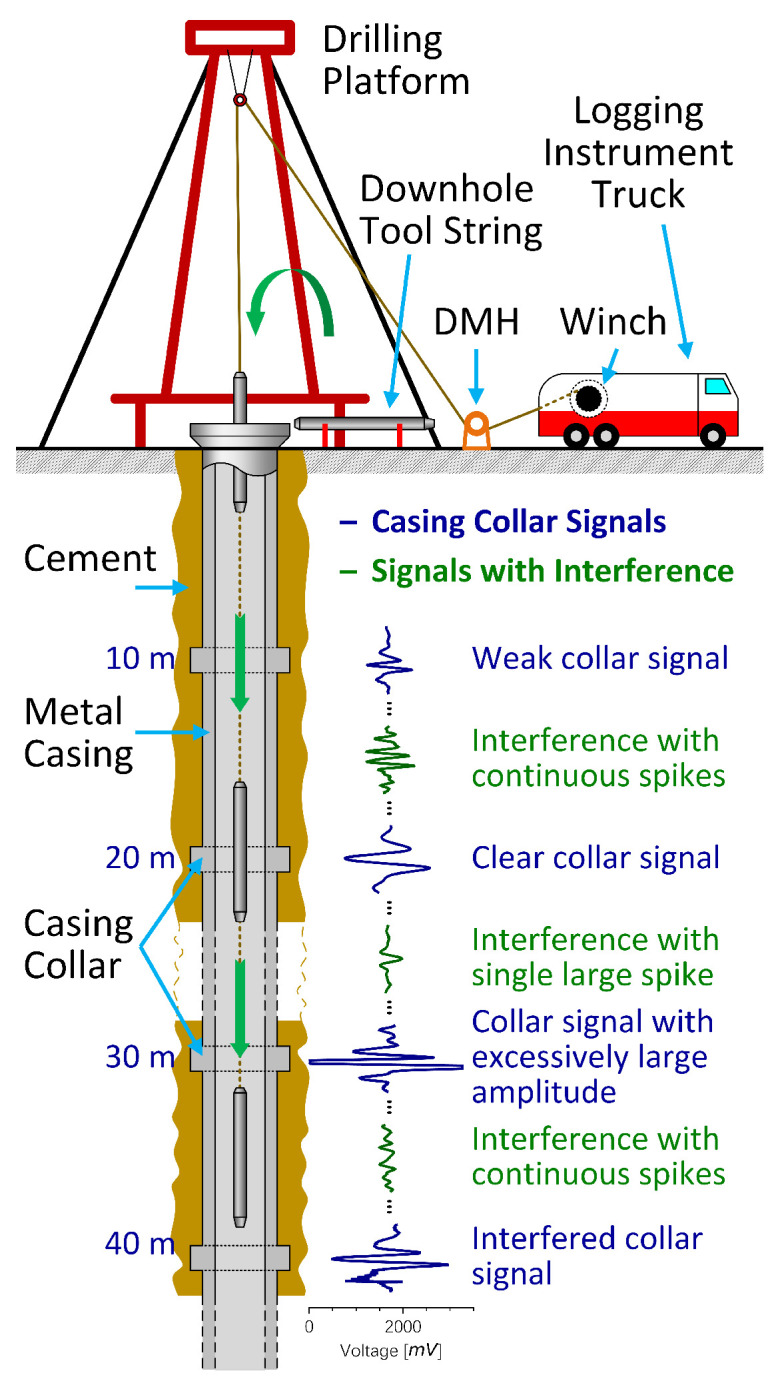
Cross-sectional illustration of a typical oil and gas well structure. Representative casing collar signatures from magnetic response are illustrated in dark blue near the corresponding casing collar, while typical interference signals are illustrated in dark green. Organized from [2].

Accurately positioning downhole toolstrings (including perforating guns, bridge plugs, and packers) is essential in modern oil and gas well operations, directly affecting maximum productivity and operational safety [1]. Central to this task is precise downhole depth measurement, a challenge compounded by the extreme geometries of wellbores, as shown in Figure 1 organized from [2], which often span thousands of meters in length while maintaining diameters of only a few inches [3].

While surface wheel measurement (SWM) method offers a cost-effective means of estimating depth via a depth measuring head (DMH) as the toolstring descends during wireline intervention operations, it is susceptible to errors induced by cable slippage and elastic stretch. Furthermore, SWM is inapplicable to emerging operations such as wireless perforating. Consequently, to achieve accurate depth measurement in the absence of a DMH, depth correction using a casing collar locator (CCL, a magnetic downhole positioning tool that detects magnetic anomalies at the casing collars of wellbores or pipes) is fundamental [4]. The CCL produces a characteristic magnetic response as an electrical signal when passing through each collar [4,5,6], termed a “CCL response” or “collar (magnetic) signature”. The characteristic magnetic response pattern typically exhibits a bipolar signature, as illustrated by dark blue waveforms in Figure 1. Through collar correlation, which refers to tying-in CCL logs with casing tally, depth reference markers are established, enabling actual and accurate depth measurement [6,7]. The casing tally, also known as the list of collars or casing string reference depths, records the depths of casing collars and is commonly extracted from cementing quality data.

While collar correlation using CCL logs is an established method, collar signature recognition presents significant challenges. CCL signal integrity can be severely compromised by multiple factors, including cable effects, wellbore conditions [8], toolstring motion (swing or rotation), amplifier saturation, and environmental noise [7,9]. Consequently, collar signature waveforms become increasingly ambiguous, necessitating robust recognition of collar signatures amid various interferences, as illustrated by dark green waveforms in Figure 1.

Various signal processing techniques have been developed to identify collar signatures under interference conditions. Traditional methods include fixed or dynamic thresholding [2,10], digital filters and template-based cross-correlation [11], time–frequency domain techniques such as Fourier or wavelet transforms [12], and physical plausibility filters [2,4]. However, these approaches exhibit limited generalizability [9,13]. With the emergence of machine learning, researchers have increasingly employed deep neural networks to automate collar signature recognition. These developments include convolutional neural networks (CNNs) and long short-term memory networks (LSTMs) [14,15,16], Additionally, advanced architectures such as transformer models [17] and physics-informed neural networks (PINNs) [18] have been proposed for related fields such as downhole signal classification, anomaly detection, and denoising tasks [13,19,20,21,22].

Nevertheless, significant challenges persist. Neural networks require substantial volumes of labeled training data, which are often unavailable or difficult to obtain in downhole environments [14,23]. This scarcity means that the available data for training collar recognition neural networks are considerably less than those for other tasks such as face recognition or image classification. Therefore, efficiently utilization of existing data is indispensable.

The limited sample amount and unique characteristics of CCL log data necessitate specialized preprocessing methods for neural network training. However, research on preprocessing methods remain scarce, despite extensive work on collar signature recognition [4,9,11,12,13,14,17,18,19]. Fortunately, extensive work exists on data augmentation for preventing overfitting and improving generalizability when training on small datasets. Data normalization methods—including min–max scaling, Z-score normalization (standardization), and robust scaling—demonstrate important roles in stabilizing input distributions and gradients, preventing vanishing and exploding gradients, and ultimately accelerating training convergence [24,25,26,27]. Label smoothing regularization (LSR) and label distribution smoothing (LDS) discourage overconfident predictions, thereby enhancing model generalizability [28,29]. Similarly, employing probability maps for boundary prediction instead of one-hot encoding (OHE) labels through boundary probabilization transforms the training objective from single-point prediction to probability distribution estimation, enabling models learn smooth and fuzzy decision boundaries that are more resistant to noise or perturbations [15,28,30]. Notably, Gaussian kernel often deliver optimal results in LDS applications [28]. Furthermore, graph augmentation techniques including randomly cropping, scaling, translation, and noise injection expand training datasets while improving model generalizability and robustness [31,32].

**Figure 2:**
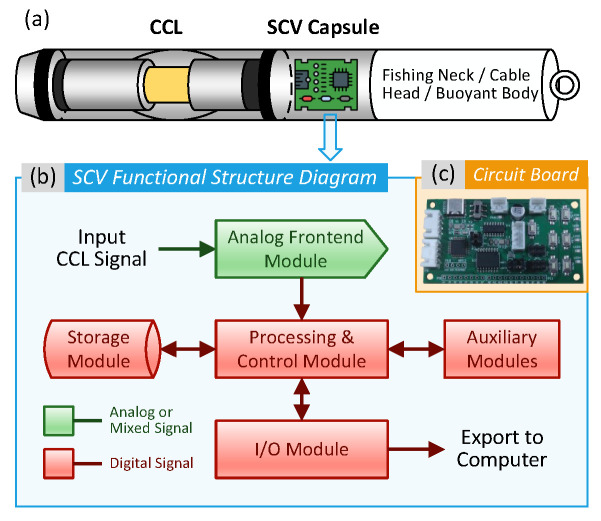
Structure of downhole toolstring integrated with the Signal Collection Vehicle (SCV): (**a**) schematic diagram of the internal structure of the perforating gun employed in this work; (**b**) functional structure diagram of the SCV; and (**c**) a SCV circuit board.

**Figure 3:**
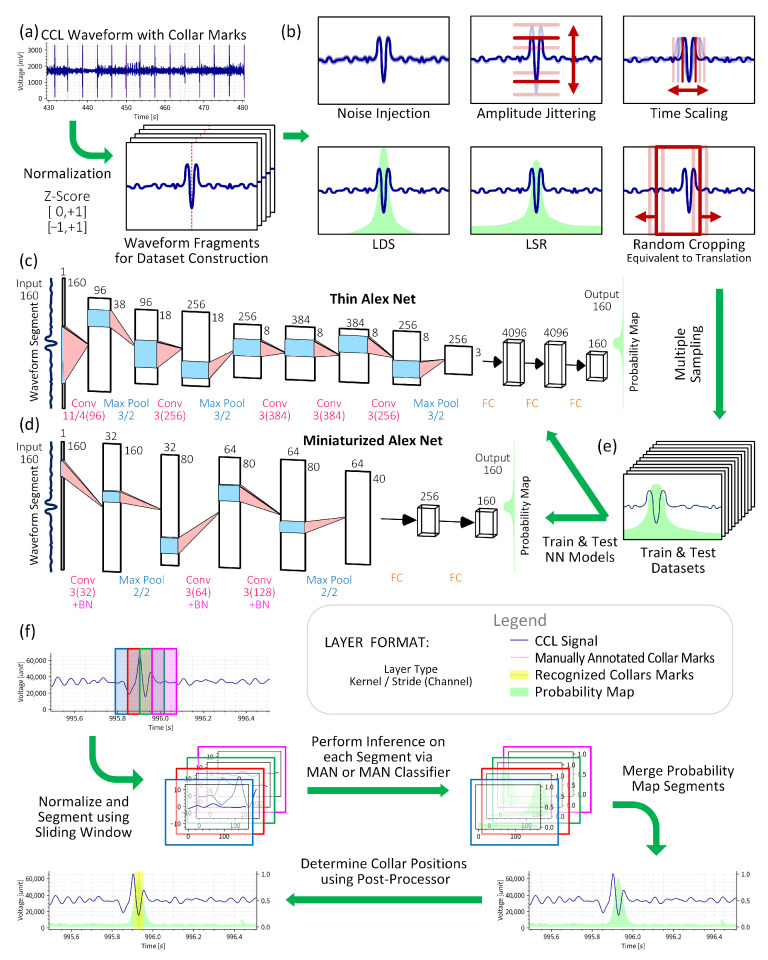
The train and inference process of this work: (**a**) fragment the normalized CCL waveform based on manually labeled collar marks, each fragment containing a collar mark at its center (waveform shapes in boxes are illustrative only); (**b**) augmentation methods for preprocessing waveform fragments and their labels; (**c**,**d**) baseline neural network architectures employed in this work; (**e**) multiple random augmentations applied to each fragment to generate diverse sub-samples variants for training and testing datasets; and (**f**) the procedure of casing collar recognition from CCL waveforms using sliding windows with overlap.

The main contributions of this paper are as follows:

- We develop a system integrated into downhole toolstring, called the Signal Collecting Vessel (SCV), illustrated in Figure 2. The SCV samples raw CCL signals downhole and converts them to digital format, and stores them as waveforms for dataset construction.
- We propose two neural networks models for collar signature recognition that serve as baselines for evaluating data preprocessing methods, as illustrated in Figure 3. The first model, Thin AlexNet (TAN), is modified from AlexNet—a classic and proven architecture in pattern recognition. The second model, Miniaturized AlexNet (MAN), is a simplified version of TAN with fewer layers.
- We propose several data augmentation methods for preprocessing original waveforms to enhance model training performance, including normalization, label distribution smoothing (LDS), label smoothing regularization (LSR), time scaling, cropping and translation, amplitude jittering, noise injection, and multiple sampling.
- We conduct extensive experiments across various configuration combinations with filed CCL logs to validate our methods. Results demonstrate that standardization, LDS, and random cropping are fundamental requirements for models training, while LSR, time scaling, and multiple sampling significantly enhance model generalization capability.

## 2. Methods

### 2.1. Problem Transformation

As previously discussed, accurate downhole toolstring positioning via collar correlation relies on the correct identification of bipolar patterns (i.e., collar signatures) within CCL logs. The centroid of each bipolar pattern is typically designated as the instant the CCL coincides with a casing collar. In the absence of DMH assistance (specifically, without the depth indexing commonly utilized in wireline logging), time-series CCL logging becomes the requisite approach.

Field practice demonstrates that collar signatures are identifiable primarily through local waveform characteristics in the vicinity of the collar, rendering distant signal features negligible. Consequently, analyzing CCL log fragments via an appropriately sized sliding window is an effective strategy, aligning with methodologies proposed in [15,16]. Furthermore, when raw CCL signals are sampled at a fixed frequency, absolute timestamps within the sliding windows become redundant; that is, the raw waveform sequence alone conveys sufficient information.

As shown in Figure 1, the casing tally, which provides a series of depths, correlates to the centroids of collar signatures (hereafter referred to as “collar marks”) on the temporal axis. The most direct representation of collar marks in CCL waveforms is one-hot encoding (OHE), where a value of 1 indicates the presence of a collar mark and 0 indicates its absence. This formulation frames collar mark prediction as a binary classification task.

However, collar marks exhibit extreme sparsity: background samples (0s) outnumber target samples (1s) by several orders of magnitude, resulting in severe class imbalance. In backpropagation neural networks (BPNNs), this imbalance leads to sparse gradients and uneven penalty distribution, causing training instability and slow convergence. Consequently, training an effective classifier becomes computationally challenging. To mitigate this, OHE is replaced by a probability map [15,33], wherein labels represent the probability of boundary occurrence. Interestingly, consistent with the theory in [33], the label reflects the distribution of relative importance across possible categories (i.e., “the presence of a collar mark”). This transformation shifts the problem from hard binary classification to boundary membership estimation, providing denser feedback during backpropagation and facilitating stable and efficient network training.

In summary, the problem of collar signature recognition is transformed into a boundary membership estimation task, where the input is a windowed temporal CCL waveform and the output is a temporal probability map.

### 2.2. Acquisition of Raw CCL Waveforms

To eliminate signal degradation associated with long cable transmission and preserve signal integrity, a Signal Collection Vehicle (SCV) was developed, as shown in Figure 2. The SCV is designed to be integrated into downhole toolstring and enable the real-time logging of raw CCL signals downhole.

The SCV comprises of the analog frontend (AFE) module, the signal processing and control module, the data storage module, the input–output (I/O) module, and auxiliary modules. As the SCV is lowered with downhole toolstring, the AFE module samples raw CCL signal at a sampling rate of 1 kHz via a 16-bit resolution analog-to-digital converter (ADC). The resulting digital data is recorded in the storage module. Upon completion of downhole operations, the SCV is salvaged, and data is exported via the I/O module.

The raw temporal CCL logs acquired by the SCV are designated as “original CCL waveforms”. Typically, a single log contains approximately 50 to 200 collar signatures, corresponding to a well depth ranging from 500 m to 2 km.

### 2.3. Dataset Construction and Augmentation

Collar marks within the field-acquired original CCL waveforms were manually annotated through expert analysis. To construct the dataset, waveforms surrounding each collar mark were fragmented into fixed-length fragments, while non-informative sections distant from the collars were excluded. As illustrated in Figure 3a, each fragment is centered on a collar mark, which is initially represented using one-hot encoding.

To enhance training effectiveness, maximize data utilization, and avoid overfitting, we present various data augmentation methods for preprocessing original CCL data. These methods encompass data normalization, regularization, transformation, and multiple sampling, which can be applied independently or in combination, as illustrated in Figure 3b.

After preprocessing, the augmented waveform fragments and target labels constitute the datasets used for model training and evaluation.

#### 2.3.1. Normalization of Waveforms

The original CCL waveform represents raw data from the ADC in unsigned integer format. Theoretically, raw data requires normalization to enhance training performance, particularly convergence speed [34]. Both min–max scaling and z-score normalization warrant investigation. For min–max scaling, waveforms are transformed to either [0, 1] or [−1, 1]. The minimum and maximum values derive from either the waveform’s dynamic range or the ADC specifications.

#### 2.3.2. Label Distribution Smoothing (LDS)

Convolving a kernel with the empirical density distribution produces a kernel-smoothed version. The effective label density distribution is defined as [28]:

(1) $$p^{\prime }\left(y^{\prime }\right)≜\int_{Y}k\left(y,y^{\prime }\right)p\left(y\right)\;dy$$

where p(y) is the label of *y* in the training data; $p^{\prime }$ is the effective density of label $y^{\prime }$; and $k(y,y^{\prime })$ is the kernel function.

One-hot encoding of collars along the timeline constitutes a validity probability distribution that proves challenging for training. Convolution smooths these hard labels, enabling each label in the validity probability distribution to incorporate information from neighboring labels [28].

While Gaussian kernels reportedly achieve optimal results among all kernel types [28], other research cautions that Gaussian assumptions may not accommodate complex real-world datasets [33]. This work employs a Gaussian kernel is employed, yielding the following label formulation:

(2) $$p^{\prime }\left(t\right)=\left\{\begin{array}{cc} \sum_{i}\sum_{t}e^{-\frac{{\left(t-t_{i}\right)}^{2}}{2\sigma^{2}}} & ,\;if<1\\ 1 & ,\;otherwise \end{array}\right.$$

where σ is the Gaussian root mean square (RMS) width; and $t_{i}$ is the moment when the *i*^th^ collar occurs.

#### 2.3.3. Label Smoothing Regularization (LSR)

LSR softens labels by redistributing a small probability portion from the correct class evenly among all classes. The distribution relationship follows [29]:

(3) $$\begin{array}{cc} p(k) & =\delta_{k,i}\\ p^{\prime }\left(k\right) & =\left(1-\epsilon \right)\delta_{k,i}+\frac{\epsilon }{K}\\ \delta_{k,i} & =\left\{\begin{array}{cc} 1 & ,\;k=i\\ 0 & ,\;k\neq i \end{array}\right. \end{array}$$

where p(k) is the ground-truth distribution over the *k*^th^ class; $p^{\prime }\left(k\right)$ is the training distribution over the *k*^th^ class; *i* denotes the correct class; *K* is the total number of classes; δ is the Kronecker delta function; and ϵ is the smoothing parameter.

When employing the sigmoid function, the parameter ϵ effectively constrains the magnitude of the output logits. This mechanism discourages overconfident predictions by preventing the largest logit from becoming disproportionately larger than others, thereby regularizing the model and enhancing its generalization capability [29]. In this study, given K=2, the training distribution simplifies to

(4) $$p^{\prime }\left(k\right)=\left(1-\epsilon \right)p\left(k\right)+\frac{\epsilon }{2}$$

where an ϵ value of 0.1 is recommended to strike a balance between accuracy and generalization capability.

#### 2.3.4. Geometric Transformations

The dataset undergoes expansion through various geometric transformations, as illustrated in Figure 3b. Transformations are employed as follows:

- Time Scaling: Waveform fragments are scaled along the time axis by random factors and subsequently resampled to restore the original sampling rate. The resampling process employs Hann-windowed sinc interpolation, the default resampling method in the TorchAudio library, to mitigate spectral artifacts, including ringing and aliasing, while ensuring effective high-frequency attenuation.
- Randomly Cropping and Translation: These transformations waveform fragments into sub-samples that match both the sliding window length and the neural network model’s input length.
- Amplitude Jittering: Waveform fragments are multiplied by random gain factors to enhance the model’s robustness and generalization capability.
- Noise Injection: Gaussian noise is added to original fragments to improve model robustness against noise.
- Flipping: Voltage or time axis flipping is excluded as such transformations would violate the physical principles governing CCL magnetic response.

#### 2.3.5. Multiple Sampling

To maximize dataset utilization, each fragment undergoes the multiple random augmentation process to generate diverse sub-samples variants, as illustrated in Figure 3e. This approach theoretically accelerates convergence and enhance augmentation effectiveness.

### 2.4. Neural Network

The proposed model, Thin AlexNet (TAN), is a time-series version of the classical AlexNet architecture. The input and output dimensions are reduced from 2D to 1D to adapt CCL waveforms. Since AlexNet has demonstrated success in image classification [31,32] and possesses sufficient simplicity to clearly demonstrate the effects of data augmentation, the TAN model serves as an appropriate baseline.

The TAN model comprises five convolutional layers with ReLU activation, three max pooling layers, and three fully connected (FC) layers, with ReLU activation applied to the first two fully connected layers, as illustrated in Figure 3c. The input of model is a fixed-length segment from a waveform, and the output produces a series of logits representing scores of collar mark classification for each temporal position within the input segments. This differs from the original AlexNet. Probability for each temporal position is obtained through sigmoid function.

Based on the TAN model, we proposed a simplified version, Miniaturized AlexNet (MAN), as illustrated in Figure 3d. MAN contains fewer convolutional layers, max pooling layers, and fully connected layers than TAN. Additionally, batch normalization (BN) layers are appended in MAN to improve training stability. MAN employs the same input–output format as TAN.

## 3. Experiments and Results

### 3.1. Evaluation Measures

To evaluate the classifiers implemented by TAN and MAN, which output probability distributions for classification, metrics that measure distance and similarity between predicted and label distributions are appropriate [33]. F1 score and cross-entropy (CE) represent standard choices for such evaluation. Additionally, the area under the precision–recall curve (AUC-PR) provides objective performance assessment for binary classifiers.

To recognize collar signatures from normalized long CCL waveforms, a sliding window of width *W* with a strid of ⌊W/2⌋ is employed. This configuration produces 50% overlap between consecutive windows, ensuring that each overlapping region is captured by exactly two adjacent windows. The window advances through the waveform in half-width increments, balancing computational efficiency with temporal resolution while maintaining analytical continuity. The probability map segments inferenced from overlapping regions of adjacent windows are averaged to produce the complete probability map, as illustrated in Figure 3f.

Through sliding window progression, the complete probability map for the entire CCL waveform is generated. Continuous intervals with probabilities exceeding the threshold are identified as valid regions, with their center positions designated as collar marks. This procedure constitutes the “post-processing” stage. The complete casing collar recognition process from waveform comprises a neural network-based classifier and a post-processor for probability map analysis, as illustrated in Figure 3f. Collar recognition performance is evaluated by comparing recognized collar positions with manually annotated reference positions. Recognized collars within the neighborhood of annotated collars are classified as true positives, while those elsewhere are classified as false positives. Missed collars are considered as false negatives. Precision, recall and F1 score are calculated for evaluation.

### 3.2. Training and Validation

The CCL waveforms utilized in the experiments were acquired from field operations in Sichuan Province, China, ensuring that the results are representative of actual downhole conditions. The dataset was partitioned into training and validation subsets using a 3:1 ratio based on the CCL logs, yielding 288 and 50 original waveform fragments, respectively. To mitigate the limitations (including funds) imposed by the restricted dataset size, a multiple sampling strategy was employed to augment the training set. The training and validation procedures were conducted offline on a workstation equipped with an AMD Ryzen 3960X CPU and dual NVIDIA RTX 3080 GPUs, utilizing the PyTorch 2.0.1 framework.

**Figure 4:**
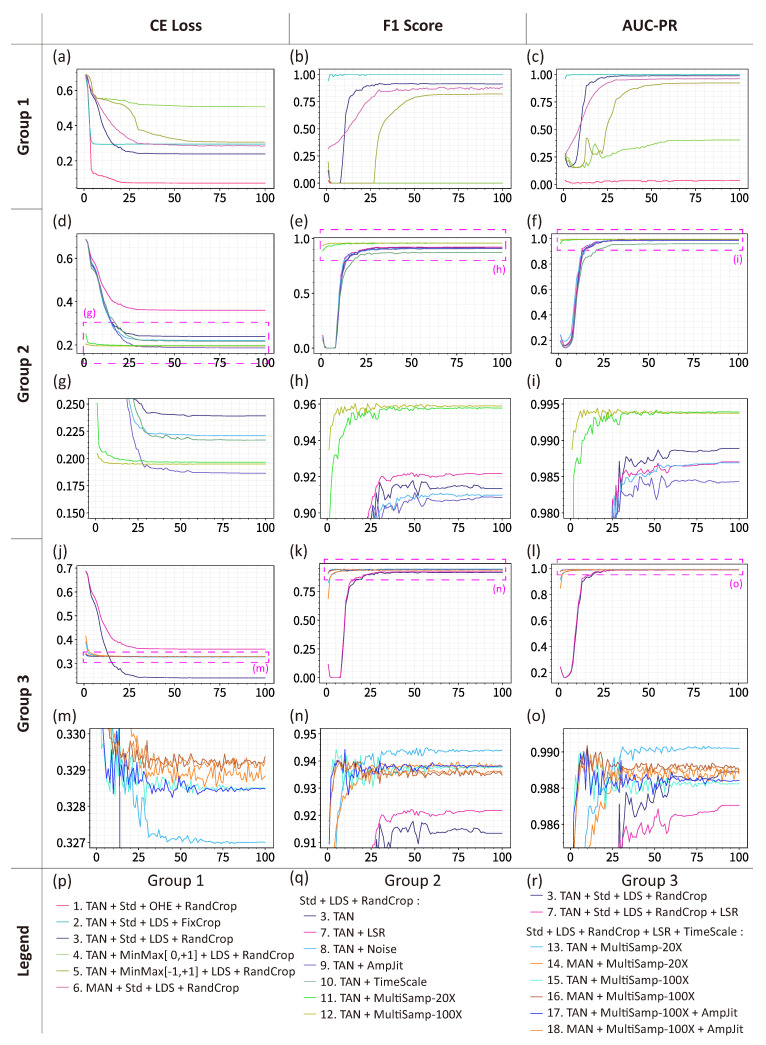
Evaluation metrics of training progress under different configurations, including cross-entropy loss, F1 score, and area under the precision–recall curve (AUC-PR). The curve indexes correspond to configurations in Table 1 and Table 2. The meanings of abbreviations refer to Table 1 and Table 2. (**a**–**c**) Evaluation metrics for Group 1 configurations. (**d**–**f**) Evaluation metrics for Group 2 configurations. (**g**–**i**) Enlarged sections of (**d**–**f**). (**j**–**l**) Evaluation metrics for Group 3 configurations. (**m**–**o**) Enlarged sections of (**j**–**l**). (**p**–**r**) Legends for (**a**–**c**), (**d**–**i**), and (**j**–**o**), respectively.

The models are trained with a batch size of 16 for 100 epochs utilizing cross-entropy loss (CE loss) with an adaptive moment estimation (Adam) optimizer. Key configurations (Cfgs.) are presented in Table 1 and Table 2. The training progress and the optimal model performance for each configuration are illustrated in Figure 4. Additionally, model inference and post-processing were evaluated on two full-length waveforms characterized by mild and moderate interference, respectively. The mild interference waveform contains 52 collars with maximum depth of 512.2 m, while the moderate interference waveform contains 77 collars with maximum depth of 771.1 m. Crucially, the waveform fragments from these evaluation waveforms were excluded from the training set. The precision, recall, and F1 score results are tabulated in Table 1 and Table 2, with the aggregated results for both waveforms are tabulated in Table 3.

### 3.3. Results and Analysis

To explore the validity and importance of augmentation methods, several real and complete waveforms are experimented to verify the models’ ability of predicting collar positions. The experimental results are indexed and divided into three groups, as illustrated in Figure 4, with curve indexes corresponding to those in Table 1 and Table 2. The first group (Cfgs. 1–6) examines normalization methods, label distribution methods, and cropping methods. The second group (Cfgs. 7–12) investigates label smoothing, noise injection, amplitude jittering, and time scaling. The final group (Cfgs. 13–18) explores optimal combination configurations.

#### 3.3.1. Fundamental Preprocessing Requirements

**Figure 5:**
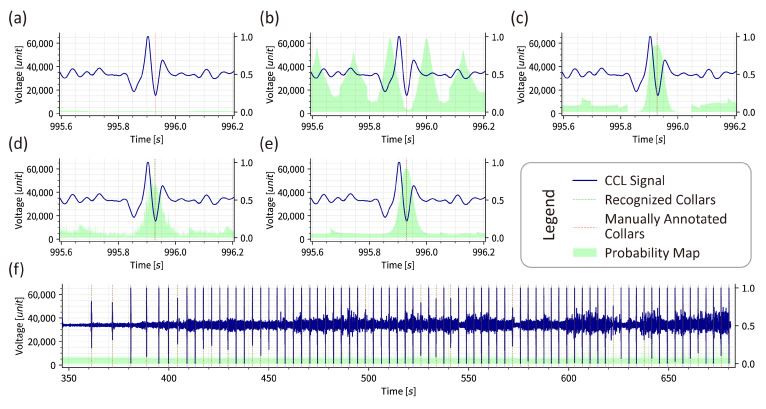
Probability maps and recognition results for various configurations. (**a**) Cfg. 1 (using OHE); (**b**) Cfg. 2 (using fixed cropping); (**c**) Cfg. 3 (using LDS and random cropping); (**d**) Cfg. 6 (using fundamental methods with MAN model); (**e**) Cfg. 13 (optimal combination candidate using TAN model); and (**f**) full results of Cfg. 14 (optimal combination candidate using MAN model).

A comparison of Cfgs. 1 and 3, which differ in label distribution method, confirms that F1 score for both classifier and recognition using one-hot encoding approaches 0, despite the CE loss decreasing more rapidly than with LDS. The probability maps corroborate this observation, as illustrated in Figure 5a,c. This indicates that one-hot encoded collar labels are difficult for models to learn, as the loss function reaches a local minimum when models consistently output negative predictions, as illustrated in Figure 4a.

Similar phenomena occur with min–max normalization versus standardization (corresponding to Cfgs. 3–5), and fixed cropping versus random cropping (corresponding to Cfgs. 2 and 3), as illustrated in Figure 5b,c. Waveforms processed with min–max normalization prove challenging for model training. Furthermore, models using fixed cropping learn only sliding window positions rather than waveform characteristics.

Comparison of Cfgs. 3 and 6 clearly demonstrates that both TAN and MAN module have the ability to estimate collar positions when using LDS, standardization, and random cropping, as illustrated in Figure 5c,d. However, MAN training proceeds more slowly than TAN training, with slightly inferior performance, as MAN contains approximately half the parameters of TAN. These findings establish that LDS, standardization, and random cropping are fundamental requirements for collar recognition model training.

#### 3.3.2. Generalization Enhancement Methods

To investigate the effects of LSR, noise injection, amplitude jittering, and time scaling, Cfgs. 7–10 based on Cfg. 3 are experimented. A comparison of curves in Figure 4d–i reveals that (a) the final CE loss for Cfgs. 8–10 is lower than that for control Cfg. 3, while Cfg. 7 shows higher loss; (b) the classifier F1 scores of Cfgs. 7–9 are similar, while Cfg. 10 shows slightly lower performance; and (c) LSR convergence is slower than others. Performance comparison between Cfgs. 7–10 and control Cfg. 3, as tableted in Table 2, demonstrates that (a) LSR exhibits higher CE loss but superior F1 score in waveform evaluation, with 0.024 improvement on the moderate inference waveform; (b) although amplitude jittering and time scaling show lower F1 scores on validation set evaluation is compared to the control configuration, their waveform evaluation F1 scores are higher, with improvements of 0.012 and 0.037, respectively, on the moderate inference waveform. These results suggest that LSR enhances generalization capability at the cost of convergence speed, while amplitude jittering and time scaling also improve generalization. However, noise injection performs worse than all other configurations. Additional experiments reveal that small noise provides limited benefits to performance, while large noise impairs performance. This likely occurs because real-world waveforms inherently contain small noise, and additional large noise hinders model learning waveform characteristics.

To investigate the effects of multiple sampling, Cfgs. 11–12 based on Cfg. 3 are experimented. Configurations with multiple sampling converge substantially faster than the control configuration, as shown in Figure 4d–i, because multiple sampling increases iterations per epoch. The F1 score improves up to 0.045 in evaluation.

In summary, LSR, amplitude jittering, time scaling, and multiple sampling significantly enhance generalization capability, while noise injection provides limited benefits. By comparing Cfg. 13 and Cfg. 14 with Cfg. 3 and Cfg. 6, respectively, these data augmentation methods achieve F1 score improvements of up to 0.027 for the TAN model and 0.024 for the MAN model.

#### 3.3.3. Optimal Configuration Identification

Based on these conclusions, Cfgs. 13–18 are experimented to identify optimal combinations while comparing with Cfgs. 3, 6 and 7, as illustrated in Figure 4j–o and Figure 5c–e and tabulated in Table 2. Several noteworthy phenomena emerge from these experiments.

First, metrics of configurations with 100× multiple sampling fluctuate more dramatically than those with 20×, suggesting the need for smaller initial learning rates when applying extensive multiple sampling. Second, simultaneous use of amplitude jittering and time scaling unexpectedly reduces the performance. We hypothesize that amplitude scaling compromises standardization benefits while improving generalization, as the relative amplitude of waveforms contains critical information. Third, Cfg. 13 achieves the highest CE loss and validation set F1 score among all TAN model configurations, yet its F1 score on the moderate interference waveform does not show corresponding improvement, as illustrated in Figure 4m–o. This occurs because multiple sampling introduces more randomly preprocessed samples, enhancing generalization capability.

Notably, both full-length evaluation waveforms contain several dozens of collar signatures, effectively functioning as a small-scale evaluation set. Consequently, it is plausible to achieve near-perfect or perfect results (an F1 score of 1.0) provided the model possesses sufficient accuracy. Furthermore, in the case of waveforms with mild interference, the collar signatures exhibit greater separability, thereby facilitating the attainment of perfect identification performance.

Most significantly, MAN performance does not degrade substantially compared to TAN, and MAN exhibits less performance degradation on moderate interference waveform than TAN, despite containing approximately half the parameters, as tabulated in Table 2. These findings suggest that (a) TAN may experience overfitting in certain dimensions; (b) collar classification could potentially be achieved with more compact networks warranting future investigation; and (c) practical applications can consider the trade-off between accuracy and parameter count.

#### 3.3.4. Performance Validation and Key Findings

The results of Cfg. 14 are illustrated in Figure 5f as an example, while the details of other experiments are not repeated here. Casing collars recognized by MAN model and collars labeled manually are marked in green and red, respectively. The casing collar positions are correctly recognized and align completely with manual annotations, providing the essential foundation for precise depth measurement.

These findings establish that the standardization, LDS, and random cropping are fundamental preprocessing requirements for collar recognition model training, while LSR, time scaling, and multiple sampling significantly enhance model generalization capability. The data augmentation methods achieve F1 score improvements of up to 0.063 for the TAN model waveform evaluation and up to 0.048 for the MAN model evaluation, compared to configurations using only fundamental methods, representing notable performance enhancements. Furthermore, the baseline models TAN and MAN exhibit performance comparable to prior works, as tabulated in Table 3. However, applying the proposed data augmentation methods yields F1 score improvements of up to 0.045 for the TAN model and 0.057 for the MAN model relative to other approaches. This observation demonstrates that the proposed data augmentation methods are effective even when the underlying model architecture is not optimal.

## 4. Conclusions

This work developed the SCV to acquire raw downhole CCL signals for dataset construction. The contributions of various data augmentation methods were systematically analyzed through baseline evaluations using the proposed neural network recognition methodology. Experimental validation with field CCL logs confirms the effectiveness and validity of proposed approaches. Results indicate that standardization, LDS, and random cropping are fundamental preprocessing prerequisites for training collar recognition models, while LSR, time scaling, and multiple sampling significantly enhance model generalization capabilities. Incorporating the proposed augmentation methods into the two baseline models results in maximum F1 score improvements of 0.027 and 0.024 for the TAN and MAN models, respectively. Furthermore, applying these techniques yields F1 score gains of up to 0.045 for the TAN model and 0.057 for the MAN model compared to prior studies. This research addresses the existing gaps in data augmentation methodologies for training casing collar recognition models under CCL data-limited conditions, and provides a technical foundation for the future automation of downhole operations.

## Figures and Tables

**Table 1:** Experimental results for various combinations of normalization, label distribution, and cropping methods.

Cfg. No.	Model	Normalization	Lbl. Dis.	Crop	Evaluation by Validation Set During Training			Evaluation by Moderate Interference Waveform			Evaluation by Mild Interference Waveform		
					CE	F1	AUC-PR	P	R	F1	P	R	F1
1	TAN	Standardization	OHE	Rand	0.0736	0	0.0350	0	0	0	0	0	0
2	TAN	Standardization	LDS	Fix	0.2943	1	0.9987	0	0	0	0	0	0
3	TAN	Standardization	LDS	Rand	0.2391	0.9134	0.9889	0.9136	0.9610	0.9367	0.9811	1	0.9905
4	TAN	MinMax [ 0, +1]	LDS	Rand	0.5074	0	0.4051	0	0	0	0	0	0
5	TAN	MinMax [−1, +1]	LDS	Rand	0.3055	0.8205	0.9234	0.0396	0.1169	0.0592	0.0335	0.1346	0.0536
6	MAN	Standardization	LDS	Rand	0.2852	0.8821	0.9619	1	0.9091	0.9524	1	1	1

All configurations use a batch size of 16, with no additional data augmentation methods applied. Configuration numbers are color-coded to match the corresponding curves in Figure 4. Abbreviations: Cfg. No. = configuration number; Lbl. Dis. = label distribution; CE = cross-entropy; F1 = F1 score; AUC-PR = area under the precision–recall curve; P = precision; R = recall; OHE = one-hot encoding; LDS = label distribution smoothing; Rand = random.

**Table 2:** Experimental results for various combinations of soft label, geometric transformations, and multiple sampling methods.

Cfg. No.	Model	Soft Label	Noise Inj.	Amp. Jit.	Time Scale	Multi. Samp.	Evaluation by Validation Set During Training			Evaluation by Moderate Interference Waveform			Evaluation by Mild Interference Waveform		
							CE	F1	AUC-PR	P	R	F1	P	R	F1
3	TAN	−	−	−	−	1	0.2391	0.9134	0.9889	0.9136	0.9610	0.9367	0.9811	1	0.9905
6	MAN	−	−	−	−	1	0.2852	0.8821	0.9619	1	0.9091	0.9524	1	1	1
7	TAN	LSR	−	−	−	1	0.3596	0.9217	0.9871	0.9610	0.9610	0.9610	0.9811	1	0.9905
8	TAN	−	+	−	−	1	0.2209	0.9098	0.9869	0.8152	0.9740	0.8876	0.9630	1	0.9811
9	TAN	−	−	+	−	1	0.1864	0.9085	0.9843	0.9367	0.9610	0.9487	0.9811	1	0.9905
10	TAN	−	−	−	+	1	0.2170	0.8726	0.9592	0.9740	0.9740	0.9740	1	1	1
11	TAN	−	−	−	−	20	0.1966	0.9577	0.9939	0.9390	1	0.9686	1	1	1
12	TAN	−	−	−	−	100	0.1949	0.9589	0.9937	0.9872	1	0.9935	1	1	1
13	TAN	LSR	−	−	−	20	0.3270	0.9439	0.9902	0.9506	1	0.9747	1	1	1
14	MAN	LSR	−	−	−	20	0.3288	0.9379	0.9888	1	1	1	0.9811	1	0.9905
15	TAN	LSR	−	−	−	100	0.3285	0.9377	0.9883	0.9625	1	0.9809	1	1	1
16	MAN	LSR	−	−	−	100	0.3294	0.9348	0.9890	0.9744	0.9870	0.9806	0.9808	0.9808	0.9808
17	TAN	LSR	−	+	−	100	0.3285	0.9381	0.9884	0.9620	0.9870	0.9744	0.9811	1	0.9905
18	MAN	LSR	−	+	−	100	0.3293	0.9355	0.9889	0.9744	0.9870	0.9806	0.9808	0.9808	0.9808

All configurations employ standardization for normalization, LDS for label distribution, random cropping, and a batch size of 16. Configuration numbers are color-coded to match the corresponding curves in Figure 4. Additional abbreviations: Inj. = injection; Amp. Jit. = amplitude jittering; Multi. Samp. = multiple sampling; LSR = label smoothing regularization; + = method applied; − = method not applied.

**Table 3:** Comparison of performance with other works.

Network	Performance					Method
	Tests	Acc	P	R	F1	
Cfg. 3	129	0.920	0.940	0.977	0.958	CCL + TAN + LDS
Cfg. 6	129	0.946	1	0.946	0.972	CCL + MAN + LDS
Cfg. 13	129	0.970	1	0.970	0.985	CCL + TAN + LDS + Data Augmentation
Cfg. 14	129	0.992	1	0.992	0.996	CCL + MAN + LDS + Data Augmentation
[14]	269	0.974	1	0.942	0.970	CCL + CNN
[14]	269	0.948	1	0.884	0.939	CCL + LSTM
[14]	269	0.978	0.959	0.991	0.975	CCL + CNN-LSTM
[2]	579	0.973	0.988	0.985	0.986	CCL + Dynamic amplitude threshold + Physical plausibility
[10]	8	1	–	–	–	CCL + Relative amplitude
[7]	–	–	–	–	–	CCL + Cross correlation + Predifined threshold
[6]	–	–	–	–	–	CCL + Wavelet transform

“–” indicates “Not mentioned”; “P”, “R” are precision and recall, respectively. Configuration numbers are color-coded to match the corresponding curves in Figure 4.

## Data Availability

The data presented in this study are available on request from the corresponding author due to confidentiality agreements with the data provider.
